# Research on epilepsy detection methods based on interpretable features and machine learning

**DOI:** 10.1371/journal.pone.0344164

**Published:** 2026-03-09

**Authors:** Yongxin Sun, Xiaojuan Chen, Xinghua Zhang, Xiaohui Cai

**Affiliations:** 1 College of Electronic Information Engineering, Changchun University of Science and Technology, Changchun, Jilin, China; 2 Baicheng Normal University, Baicheng, Jilin, China; 3 College of Science and Techology Changchun, Changchun, Jilin, China; Athinoula A Martinos Center for Biomedical Imaging, UNITED STATES OF AMERICA

## Abstract

Epilepsy is a prevalent neurological condition that impacts a significant number of individuals worldwide. Patients’ physical and mental health, as well as their daily activities, are significantly affected by seizures, necessitating prompt diagnosis and treatment. The automatic detection of epilepsy using electroencephalogram (EEG) signals has been a significant area of research. Nevertheless, the majority of current methods are based on intricate feature engineering processes that require the extraction and selection of a large number of features to identify the most discriminative feature sets. This results in a high level of algorithmic complexity, inadequate robustness, and inadequate interpretability, which complicates the provision of theoretical support to clinicians. This paper proposes a pathophysiology-driven, interpretable machine learning algorithm to address the limitations of current EEG-based epilepsy detection methods, which include poor interpretability and complex feature engineering. We developed a low-dimensional, interpretable feature combination consisting of only five features and systematically validated its discriminative capability across various epilepsy phases by innovatively integrating electrophysiological markers of epileptic seizures with nonlinear dynamical properties. In the binary classification of seizure versus non-seizure EEG segments, the XGB classifier achieved the highest accuracy of 98.73% and an F1 score of 98.57%. Classification accuracy for interictal versus ictal periods reached 95.33%, with an F1 score of 95.27%. In the challenging ternary classification task encompassing preictal, interictal, and ictal periods, the model achieved a respectable accuracy of 86.3% and an F1 score of 85.79%. Cross-database validation yielded a maximum accuracy of 82.17% and an F1 score of 81.99%, confirming the proposed features’ robust generalization capability and transformative potential. This feature set exhibits outstanding and stable performance across all models, as demonstrated by evaluations across two public datasets using five machine learning classifiers. In addition, SHAP values quantified the contribution of each feature to predictions, thereby providing a transparent decision-making rationale that substantially improves the algorithm’s interpretability and clinical utility.

## 1. Introduction

Epilepsy is a prevalent neurological condition affecting approximately 70 million individuals worldwide, spanning all age groups [1]. The onset of epilepsy results from transient neurological dysfunction caused by abnormal neuronal discharges, characterized by suddenness, irregularity, and recurrence. Prolonged epilepsy can lead to memory loss and cognitive decline, significantly impacting patients’ daily lives and mental health [2]. However, according to the World Health Organization, it is estimated that 70% of epilepsy patients can achieve seizure freedom if accurately diagnosed and treated. Therefore, the treatment of epilepsy relies on the precise detection and diagnosis of the condition, underscoring its critical importance.

In 1924, Hans Berger, a German neuropsychiatrist, pioneered the recording of the human electroencephalogram (EEG) using precision instruments, thereby establishing a firm foundation for subsequent neuroscience research and opening new avenues. This was made possible by the advancement of science and technology. The International League Against Epilepsy (ILAE) confirmed the significant changes in human EEG during seizures in 1981, thereby providing an essential and objective basis for the diagnosis of epilepsy, based on a number of clinical observations and experimental studies. The objective premise for the diagnosis of epilepsy is provided by the significant changes in human brain electrical signals that occur during a seizure. Epileptic EEG signals can be classified into various stages, including preictal, ictal, and interictal, based on the duration of the seizure. Preictal period: The initial few minutes to tens of minutes of a seizure. The timely detection and identification of EEG signals during this period can enable the prediction of epilepsy and provide the patient with the necessary time to prepare for any additional psychological and physical injuries that may result from epileptic seizures. Ictal period: The period of epileptic seizures. Interictal period: The asymptomatic interval between two clinical epileptic seizures is termed the interictal period. During this phase, the patient’s clinical symptoms have resolved, though their electroencephalogram may still reveal abnormal discharges [3]. The formulation of a treatment plan for epilepsy is facilitated by the precise identification of the aforementioned periods.

Currently, physicians collect patients’ electroencephalogram (EEG) signals by standardizing the placement of multiple electrodes on the patient’s head. The resulting EEG data is typically large in volume [4], which makes the analysis time-consuming and inefficient, relying heavily on the subjective judgment of the physician without objective evidence. Therefore, automated epilepsy detection based on machine learning has become a primary research direction in epilepsy detection [5–7]. Machine learning-based automated epilepsy detection methods generally include steps such as EEG signal preprocessing, feature extraction, feature selection, and classifier design [8]. Among these, feature extraction is a critical link that directly affects the performance of the classifier. However, existing feature extraction methods often struggle to comprehensively represent the complex characteristics of epileptic EEG signals when extracting a single type of feature, limiting further improvements in detection performance. On the other hand, extracting a large number of features requires feature selection for filtering, which increases complexity and introduces bias, lacking generalizability. Furthermore, traditional EEG analysis methods still face limitations in the interpretability and effectiveness verification of features [9], making it difficult to fully mine the discriminative features of EEG signals and explain their intrinsic association with seizures, which is not referenced by physicians. To address this issue, this paper conducts a systematic study on the effectiveness and interpretability of features related to epileptic EEG signals, proposing a set of features that comprehensively represent the neurophysiological patterns of epileptic seizures for the purpose of epilepsy detection. Additionally, the effectiveness and generalizability of the proposed features are verified from the perspectives of model performance and interpretability. The main contributions of this paper are as follows:

1. We propose a framework for interpretable features that integrates clinical a priori knowledge. In contrast to conventional approaches that depend on high-dimensional, intricate features, we systematically developed a low-dimensional feature set consisting of only five features grounded in the pathophysiological principles of epilepsy. The features exhibit computational efficiency and demonstrate clear clinical significance, thereby enhancing the interpretability of results.
2. We confirmed the outstanding generalization and robustness of the proposed feature combination: Comprehensive evaluations were performed on two public datasets utilizing five different machine learning classifiers. Experimental results indicate exceptional and consistent performance across all models, confirming the inherent discriminative power and robustness of the feature combination, independent of specific models, thereby ensuring reliability for clinical deployment.
3. Developed a comprehensive interpretability framework encompassing features and models. In addition to providing high-accuracy models, we assessed the predictive decisions of the optimal XGBoost model through SHAP analysis. This method elucidates the contribution and directionality of each feature to the final classification outcome, transforming the model’s decisions into evidence that clinicians can understand. This offers essential theoretical backing for supplementary diagnosis.

## 2. Related work

The core principle of epilepsy detection lies in the in-depth analysis of the significant differences between electroencephalogram (EEG) signals during seizures and those in a normal state. These differences are primarily reflected in multiple dimensions such as frequency, amplitude, and waveform complexity. EEG signals during epileptic seizures typically exhibit characteristic patterns such as abnormally high-amplitude spikes, sharp waves, polyspikes, and rhythmic discharges, whereas normal EEG signals display relatively stable physiological rhythms. The reasonable extraction of EEG signal features is fundamental to epilepsy detection. Existing research has explored various feature extraction methods, including time-domain analysis, frequency-domain analysis, time-frequency analysis, and nonlinear dynamics analysis [10–12].

Slimen et al. [13] calculated the number of spikes in EEG signals during three states: interictal, preictal, and ictal. The results indicated that this metric gradually increases with the progression of the epileptic seizure. Furthermore, the average spike rate during the preictal period showed a significant statistical difference compared to the interictal period.

Chen et al. [14] proposed an algorithm for seizure detection based on the time-frequency domain and nonlinear features. They extracted mixed features, including Approximate Entropy (ApEn), Fuzzy Entropy (FuzzyEn), Sample Entropy (SampEn), and Standard Deviation (STD), from the subbands decomposed by Discrete Wavelet Transform (DWT) to form a feature set. After selecting features using the Random Forest algorithm, they employed three classifiers for classification, achieving impressive detection results on both the BONN dataset and the New Delhi dataset. Al-Hadeethi et al. [15] constructed feature vectors using a set of statistical features, implemented a two-level feature selection strategy for feature ranking, and employed an AB-BP-NN network for classification. Qureshi et al. [16] proposed a fuzzy-based model for epilepsy seizure detection, which integrates feature extraction, feature selection, and a fuzzy classifier to accomplish the task of epilepsy detection. Wang et al. [17] proposed an epilepsy detection network combining a convolutional neural network (CNN) and a long short-term memory (LSTM) network to extract EEG signal features from multiple perspectives to improve the accuracy of recognition. Xin Xu et al. [18] proposed a method based on nonlinear features of electroencephalogram (EEG) signals and Gradient Boosting Decision Trees (GBDT) to identify seizure and non-seizure periods. Through ten-fold cross-validation, an average recognition accuracy of 91.76% was achieved. Zhuohan Wang et al. [19] utilized Continuous Wavelet Transform (CWT) to generate phase spectrum and power spectrum of electroencephalogram (EEG), which were then fed into the designed branches of Convolutional Neural Network (CNN) and Vision Transformer (ViT) to extract more discriminative EEG features, achieving an accuracy rate of 98.45%. Hany F. Atlam et al. [20] proposed a hybrid feature selection method that combines Principal Component Analysis (PCA) and Discrete Wavelet Transform (DWT) to extract both time-domain and frequency-domain features. This method, when integrated with Support Vector Machine (SVM), achieved an accuracy of 97.03% in epilepsy detection. Uddipan Hazarika et al. [21] utilized the Hurst exponent and discrete wavelet transform methods for feature extraction, determining the effectiveness of the features. They validated their findings using various machine learning algorithms, achieving satisfactory identification results. Hussain et al. [22] extracted Hurst exponent (HFD) and Katz fractal dimension features using adaptive rate Discrete Wavelet Transform (DWT), and implemented epilepsy detection in combination with Support Vector Machine (SVM). Hadiyoso et al. [23] achieved classification recognition of EEG signals at different times of epilepsy by selecting a feature set composed of spectral entropy, Katz, and Sevick fractal dimensions, in conjunction with a Naive Bayes (NB) classifier. Shiqi Liu et al. [24] applied correlation coefficient and distance correlation feature selection methods to effectively implement epilepsy detection across different datasets using the combination of STD and FD-NL with bagged tree classifiers.

Although the aforementioned feature extraction-based methods for epilepsy detection have achieved good detection performance, they often require the extraction of a large number of features and the use of specific feature selection methods to identify effective features. This not only increases the complexity of the detection task but also results in poor interpretability and generalization ability. Considering the accuracy of epilepsy detection, computational complexity, and the acceptance by physicians, this paper proposes a set of interpretable and highly generalizable features. When combined with machine learning algorithms, these features demonstrate superior results in recognizing different epilepsy phases across two public datasets.

## 3. Materials and methods

**Fig 1:**
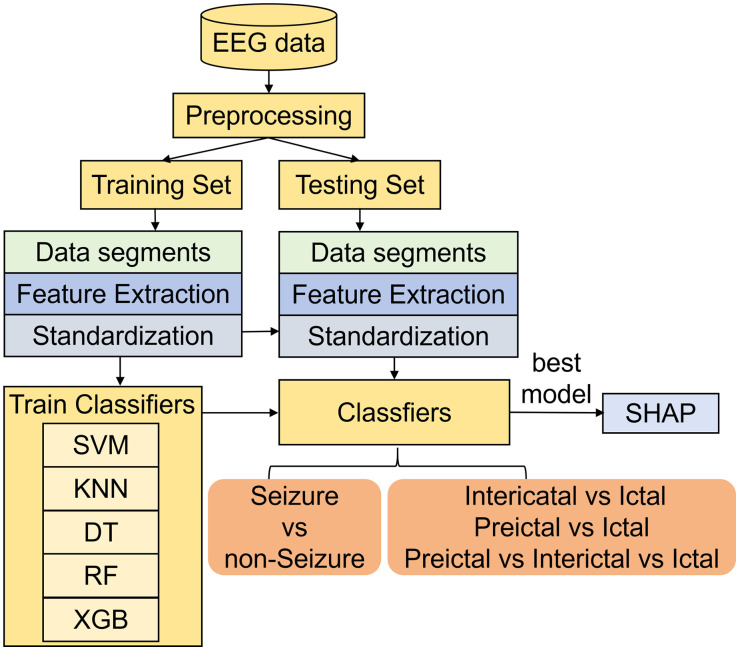
Workflow diagram of the epilepsy seizure detection system architecture.

Fig 1 illustrates the workflow of the proposed epilepsy seizure detection system architecture. The following sections provide a detailed discussion of each module.

### 3.1. Data sets

This study utilized two publicly available datasets, the Bonn University (BONN) EEG dataset for epilepsy and the New Delhi Sleep Center (NDSC) EEG dataset for epilepsy.

The Bonn Epilepsy EEG Dataset is a commonly used benchmark dataset for distinguishing normal from abnormal brain signals [25]. This dataset comprises five subsets labeled A, B, C, D, and E. Subset A and B contain scalp EEG data from healthy subjects; subsets C and D contain intracranial EEG data from epilepsy patients during non-seizure periods; while Subset E contains intracranial EEG recordings acquired during epileptic seizures. Each subset comprises 100 single-channel EEG segments, each lasting 23.6 seconds with a sampling frequency of 173.6 Hz. This study designates these five subsets as A, B, C, D, and E, respectively. This dataset has been extensively utilized in numerous EEG signal analysis studies.

**Fig 2:**
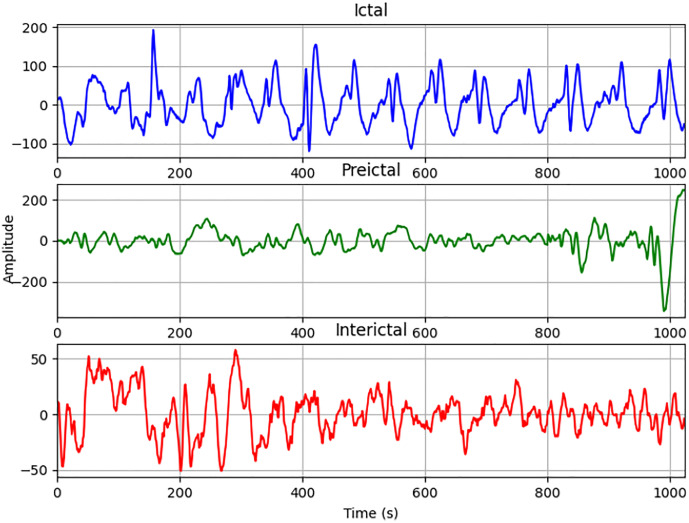
Visualization of EEG signals for three types of epilepsy.

The NDSC dataset was used to classify preictal, interictal, and ictal EEG data [26]. Data were collected from ten epilepsy patients at the Hauz Khas Neuro and Sleep Center in New Delhi. During acquisition, gold-plated scalp EEG electrodes were placed according to the 10–20 electrode placement scheme, and data were recorded at a 200 Hz sampling rate using the Grass Telefactor Comet AS40 amplification system. The collected epileptic signals were filtered within the 0.5 to 70 Hz frequency band, then segmented and categorized into three phases: preictal, interictal, and ictal. This process yielded EEG signal data for each epileptic phase required for the study. Folder names denote the seizure phase, with each folder containing 50.mat files storing EEG time-series signals. Each.mat file contains 5.12 seconds of continuous EEG signal comprising 1024 time-series data samples. Fig 2 presents the visualization of EEG signals corresponding to three distinct categories of epilepsy seizures. The horizontal axis represents the number of samples of the EEG signals, and the vertical axis represents the sample value.

### 3.2. Pretreatment

Electroencephalographic (EEG) signals are weak physiological electrical signals that are highly susceptible to external noise contamination during the acquisition process. This results in the desired EEG signals being mixed with various artifacts, which can obscure some of the information carried by the EEG signals and negatively impact subsequent analytical research. To reduce noise in the EEG signals, this study employs a method that combines Ensemble Empirical Mode Decomposition (EEMD) and Time-Frequency Peak Filtering (TFPF) for filtering the EEG signals [27], in order to enhance signal quality and improve the accuracy of subsequent feature extraction. To enhance the dataset and reduce potential information loss at segment boundaries, the continuous EEG signals were divided using a sliding window of 256 data points, with a 50% overlap between successive frames.

### 3.3. Feature extraction

#### 3.3.1. Pathological features (PLF).

In the electroencephalogram (EEG) signals of epilepsy, spike and sharp waves are two typical pathological waveforms closely associated with seizures. Spike waves usually show transient discharges of high amplitude, while sharp waves are longer in duration and lower in amplitude. The appearance of these two waveforms often signals abnormal firing activity of neurons in the brain. Therefore, extracting and analyzing the characteristics of spike and sharp waves is important for epilepsy detection.

According to the medical descriptions of spikes and sharp waves in the literature [28], spike waves are the most fundamental form of paroxysmal electroencephalographic activity, also known as transient electroencephalography, with a duration of 20–70 ms (14.5 to 50 Hz) and an amplitude exceeding 50 µV. In contrast, spikes have a time limit of 70–200 ms (5 to 14.7 Hz), with an amplitude ranging from approximately 100–200 µV. Based on the definitions above, we propose a method for extracting energy characteristics of pathological waveforms. This involves first extracting all peak information for each sample, and then calculating the corresponding wave energy based on the constraints of duration, amplitude, frequency, and other factors defined. The specific process for extracting pathological features is illustrated in Algorithm 1.

**Algorithm 1. Spike-and-sharp wave energy extraction algorithm.**

Input:

EEG sample *x*

wave-path: *{x*_*t,i*_*, y*_*t,i*_*}*_*i=1,2*_

Amplitude range: *{x*_*a,i*_*,y*_*a,i*_*}*_*i=1,2*_

Output:

Wave energy: *{E*_*i*_*}*_*i=1,2*_

1. For *i* = 1–2 do

2. Use the function find-peaks to find all local maximum points in X as a set of candidate peaks for class *i*^th^ epileptic wave: *P*_*i*_ *= {p*_*1i*_*,p*_*2i*_*,...,p*_*mi*_*}*←find-peaks(*x*,*i*)

3. Select candidate peaks where wave-path and amplitude fall within the range: {*x*_*t,i*_*, y*_*t,i*_} and {*x*_*a,i*_*,y*_*a,i*_};

4. $E_{m,i}=\sum_{x_{t,i}}^{y_{t,i}}\sum_{x_{a,i}}^{y_{a,i}}{x_{m,i}}^{\text{2}}$ ← *P*_*i*_^*wath−path∈{x*^_*t,i*_^*,y*^_*t,i*_^*},amplitude range∈{x*^_*a,i*_^*,y*^_*a,i*_^*}*^

5. End for

6. Return *E*_*i*_

Where *i* = 1 and 2, they correspond to spike waves and sharp waves, respectively. The wave-path and amplitude range are defined by their parameters, where $x_{t,i},y_{t,i}$ denote the lower and upper limits of the wave path within the specified interval, and $x_{a,i},y_{a,i}$ indicate the lower and upper limits of the amplitude within the specified interval, respectively.

#### 3.3.2. Nonlinear dynamical features (NDF).

(1) Permutation Entropy (PE): Entropy is an important indicator for measuring the degree of disorder in a system; the higher the disorder, the greater the corresponding entropy value. In recent years, an increasing number of studies have demonstrated that the brain is a highly complex chaotic system, and electroencephalographic (EEG) signals, as a typical nonlinear time series signal, exhibit significant nonlinear dynamic characteristics. Consequently, researchers have begun to widely adopt nonlinear dynamic methods for analyzing and modeling EEG signals. Among these, entropy-based features have been extensively applied in various EEG-related tasks such as sleep stage identification, fatigue state monitoring, and seizure detection and prediction, due to their effectiveness in reflecting the complexity and uncertainty of signals. Commonly used entropy measures in EEG analysis include Approximate Entropy (ApEn), Sample Entropy (SampEn), Fuzzy Entropy (FuzzyEn), and Permutation Entropy (PE). Compared to other types of entropy, Permutation Entropy has distinct advantages in terms of computational efficiency, resistance to noise interference, and data length requirements [29]. Therefore, this paper selects Permutation Entropy as the primary method for extracting nonlinear features from EEG signals to better characterize the dynamic changes of the signals.

For the original EEG sequence $X_{i}=\left\{x_{\text{1}},x_{\text{2}},\ldots x_{n}\right\}$, let the embedding dimension be m and the delay time be τ. By performing phase space reconstruction on the original signal, we obtain M reconstruction vectors, where M=n−(m−1tau. These reconstruction components can be arranged into M×m matrix, as shown in Equation (1).


$$\left[\begin{array}{cccc} @cccc@x_{\text{1}} & x_{2+\tau } & \cdots & x_{1+(m-1)\tau }\\ x_{\text{2}} & x_{2+\tau } & \cdots & x_{2+(m-1)\tau }\\ \vdots & \vdots & & \vdots \\ x_{j} & x_{j+\tau } & \cdots & x_{j+(m-1)\tau }\\ \vdots & \vdots & & \vdots \\ x_{M} & x_{M+\tau } & \cdots & x_{M+(m-1)\tau } \end{array}\right]$$ (1)

For the $j^{th}$ reconstructed component $\left\{x_{j},x_{j+\tau },\cdots ,x_{j+(m-1tau}\right\}$, the elements of the component are rearranged in ascending order, as expressed by the following equation:


$$x_{j+(j_{\text{1}}-1)\tau }\leq x_{j+(j_{\text{2}}-1)\tau }\leq \cdots \leq x_{j+(j_{m}-1)\tau }$$ (2)

In this context, $j_{\text{1}},j_{\text{2}},\cdots ,j_{m}$ represents the index of each element in the reconstructed components. Therefore, for each reconstructed component after sorting, the corresponding arrangement of indices can be obtained, and different arrangements are recorded as new symbol sequences.


$$S\left(l\right)=\left(j_{\text{1}},j_{\text{2}},\cdots ,j_{m}\right)$$ (3)

In the expression l=1,2,⋯,K, there are at most *m*! possible permutation results, denoted as K≤m!. The probability of each symbol sequence appearing is denoted as $P_{\text{1}},P_{\text{2}},\cdots ,P_{K}$. Therefore, the permutation entropy of the time series is defined as follows:


$$H_{PE}\left(m\right)=-\sum_{l=1}^{K}P_{l}lnP_{l}$$ (4)

(2) Higuchi fractal dimension (HFD): By constructing multiple sub-sequences with varying time intervals, the slope of the logarithmic length-scale curve is used to estimate the fractal dimension. Unlike traditional methods for calculating fractal dimensions, HFD directly analyzes the temporal structure of the signal, demonstrating remarkable performance in non-stationary biological signals, such as electroencephalograms (EEG). The calculation steps are as follows:
  1. Let the scale factor be k, and construct k sets of different time scale sequences for EEG signals with the following expressions:

$$x_{m}^{k}=\left\{x\left(m\right),x(m+k),x(m+2k),\cdots ,x(m+\left[\frac{N-m}{k}\right]k)\right\}(m=1,2,\cdots ,k)$$ (5)

1. Normalize each subsequence to get the following equation:

$$L_{m}\left(k\right)=\frac{\text{1}}{k}\left(\sum_{i=1}^{\left[\frac{N-m}{k}\right]}\left|x\left(m+ik\right)-x\left(m+\left(i-1\right)k\right)\right|\right)\times \frac{N-1}{\left[\frac{N-m}{k}\right]k}$$ (6)

1. The slope of the fitted logarithm of the normalized length of the subsequence with respect to the scale is the HFD value with the following formula:

ln⟨L(k)⟩∝−HFD⬝lnk (7)

(3) Dynamic Feature Increment (DFI): Dynamic feature increment refers to the first-order difference operation applied to the original feature sequence to capture the trend of changes in feature values between adjacent time windows. By normalizing, taking the absolute value, and summing the first-order differences of each feature, this method effectively enhances the sensitivity of features to state transitions without compromising the physical significance of the original features. By taking the absolute value and summing the first-order differences of the extracted pathological features, entropy features, and classification features, the dynamic intensity index of the electroencephalogram (EEG) signal is calculated. Its mathematical expression is as follows:

$$DFI=\sum \left|\overset{―}{\Delta Spike\_energy}+\overset{―}{\Delta Sharp\_energy}+\overset{―}{\Delta HFD}+\overset{―}{\Delta PE}\right|$$ (8)

In this equation, $\overset{―}{\Delta Spike\_energy}$, $\overset{―}{\Delta Sharp\_energy}$, $\overset{―}{\Delta HFD}$ and $\overset{―}{\Delta PE}$ represent the normalized values of the first-order differences of the corresponding features, respectively.

### 3.4. Machine learning based classifier algorithm

To evaluate the effectiveness of the extracted features, several classical classification algorithms are used for the experiments in this paper, including K Nearest Neighbor Algorithm (KNN), Support Vector Machine (SVM), Random Forest (RF), Decision Tree (DT), and Extreme Gradient Boosting (XGB). These classifiers have been chosen due to their excellent performances in a wide range of classification tasks and their good adaptability to different types of data distributions.

KNN algorithm is a nonparametric instance-based learning method. It calculates the distance between a new sample and each sample in the training set to find the nearest K neighbors and classifies them according to most of their categories. The KNN algorithm has a simple structure, but it shows better classification effect when the boundary between categories is more complex or irregular.

SVM is a powerful supervised learning algorithm, whose core idea is to find an optimal hyperplane so that the samples of different categories can be separated as much as possible, and the maximum classification interval is maintained. SVM performs well in high-dimensional spaces and is especially suitable for the binary classification problem where there are obvious classification boundaries.

RF is a classification method based on integrated learning, which improves the generalization ability of the model and reduces the overfitting problem by constructing multiple decision trees and combining their prediction results. The method is particularly suitable for dealing with high-dimensional datasets and can achieve high classification accuracy by aggregating the prediction results of individual trees.

DT is a basic but intuitive classification model. It performs classification by dividing the feature space into subsets based on feature values. Although Decision Tree is prone to overfitting when used alone, it provides a good benchmark for comparison with more sophisticated integration methods.

XGB is a sophisticated supervised learning algorithm derived from the gradient boosting framework. The basic principle is to combine multiple weak classifiers to create a high-performance predictive model, while improving the model’s performance by iteratively minimizing a specified loss function. The approach integrates regularization terms in the optimization process, efficiently managing model complexity and reducing overfitting, therefore improving the model’s generalization capacity. Due to its computational efficiency, exceptional scalability, and robust capacity to manage high-dimensional structured data, XGB has been extensively utilized in numerous biomedical signal categorization applications.

### 3.5. SHAP-based interpretability analysis approach

Shapley Additive exPlanations (SHAP) is a model interpretation method based on the principle of Shapley Value in cooperative game theory, which was first systematically proposed by Lundberg and Lee in 2017. The core idea of the method is to achieve a transparent explanation of the decision-making process of machine learning models by quantifying the marginal contribution of each feature to the model prediction. The advantage of the SHAP method is that it combines both model independence and theoretical rigor, and is able to provide a consistent and interpretable assessment of the importance of features at both the global and local levels. In the study of machine learning model interpretability, the SHAP method solves the problem of inconsistency in interpretation that may exist in traditional feature importance methods through a rigorous mathematical framework, and provides a reliable interpretation tool for the decision-making mechanism of complex models.

For a given input sample, the SHAP value of each feature reflects the average marginal contribution of that feature in all possible combinations with other features. Assuming that the input sample is $X_{i}=\left\{x_{i,1},x_{i,2},\ldots x_{i,j}\right\}$ and the set of features is $F=\left\{f_{\text{1}},f_{\text{2}},...,f_{n}\right\}$, for the $j^{th}$ feature $x_{i,j}$ of the $i^{th}$ sample, the computation of its SHAP value $\phi_{i,j}$ follows the definition of the Shapley value in cooperative game theory, which is mathematically expressed as follows:


$$\phi_{i,j}=\sum_{S\subseteq F\backslash \left\{f_{j}\right\}}\frac{\left|S\right|!\left(M-\left|S\right|-1\right)!}{M!}\left[g\left(S⋃\left\{f_{j}\right\}\right)-g\left(S\right)\right]$$ (9)

Where g is the trained model, S is the subset of features that does not contain feature $f_{j}$, |S| is the number of elements in the subset of features, and M is the total number of all features.

By performing a weighted average of all possible feature subsets, the Shapley value of the feature is ultimately obtained. This process ensures the fairness and completeness of feature contribution assessment. When the prediction model yields a result of ${\mathrm{\backslash stackrel}\wedge y}_{i}$ for the $i^{th}$ sample, and the model’s baseline value is $\phi_{\text{0}}$, the following equation holds:


$${\mathrm{\backslash stackrel}\wedge y}_{i}=\phi_{\text{0}}+\sum_{j=1}^{M}\phi_{i,j}=\phi_{\text{0}}+\phi_{i,1}+\phi_{i,2}+...+\phi_{i,M}$$ (10)

The application of SHAP methods in the task of epilepsy detection based on electroencephalogram (EEG) signals is of great clinical importance. Assuming that the set of EEG features includes various types, such as time domain, frequency domain, and nonlinear dynamic features, it is often difficult for traditional methods to accurately assess the relative importance of each feature to the classification results. SHAP analysis allows quantification of the contribution of each EEG feature to status epilepticus detection. This fine-grained analysis of feature contributions can help to reveal the neurophysiological mechanisms behind epileptic seizures, while also providing interpretable decision support for clinical diagnosis.

## 4. Experimental results and discussion

### 4.1. Evaluation indicators

This paper uses accuracy, precision, recall, sensitivity, and F1 score as evaluation metrics for epilepsy detection. First, it is essential to clarify four basic concepts: let TP denote the number of electroencephalogram (EEG) signals classified as epileptic and correctly predicted as epileptic samples; TN represents the number of EEG signals that are non-epileptic and correctly predicted as non-epileptic samples; FP indicates the number of EEG signals that are non-epileptic but incorrectly predicted as epileptic; FN signifies the number of EEG signals that are epileptic but incorrectly predicted as non-epileptic.

1. Accuracy (Acc): It refers to the ratio of the number of correctly predicted samples to the total number of samples, used to evaluate the basic performance of the algorithm classification. It is the most common metric for model classification evaluation, represented as follows:

$$Acc=\frac{TP+TN}{TP+TN+FP+FN}$$ (11)

1. Specificity (Spe): It refers to the proportion of non-epileptic electroencephalograms (EEGs) that are correctly predicted by the model as non-epileptic EEGs. It is used to evaluate the differences in the characteristics of various EEG states extracted by the model, reflecting the model’s ability to avoid false positives, represented as follows:

$$Spe=\frac{TN}{TN+FP}$$ (12)

1. Sensitivity (Sen): It refers to the ratio of epileptic signals correctly predicted by the model as epileptic, used to evaluate the algorithm’s sensitivity to epileptic electroencephalogram signals, represented as follows:

$$Sen=\frac{TP}{TP+FN}$$ (13)

1. Precision (Pre): It refers to the probability that samples predicted as having epilepsy are indeed actual cases of epilepsy. It is used to measure the severity of false positives, represented as follows:

$$Pre=\frac{TP}{TP+FP}$$ (14)

1. F1 Score: It refers to the harmonic mean of precision and sensitivity, taking into account both false positives and false negatives. In this paper, some datasets are imbalanced, and this metric reflects the model’s performance better than accuracy. It is represented as:

$$F1=2\times \frac{Pre\times Sen}{Pre+Sen}$$ (15)

The dataset for this study involved multiple classification tasks. We use macro-averaging to extend the accuracy (Acc), precision (Pre), sensitivity (Sen), and F1 scores from the binary classification task to the multi-category task. Specifically, we compute each evaluation metric separately for each category and then take the average of the evaluation metrics across all categories. This approach can effectively mitigate the impact of data imbalance across different categories on performance, and thus more objectively reflect the model’s recognition performance on different data types.

### 4.2. Feature validity analysis

Spike energy (SpikeE), sharp energy (SharpE), Higuchi fractal dimension (HFD), and permutation entropy (PE) were calculated for each signal fragment. In order to perform a preliminary a priori analysis of the feature distributions, we used a univariate Box-and-Whisker Plot to visualize the above features for different categories. The Box-and-Whisker Plot can visualize the concentration trend and the degree of dispersion of the data, and effectively identify potential outliers. In the plot, the distribution includes the maximum, minimum, mean, and outliers of the data, and the outliers in the distribution are indicated by circles.

**Fig 3:**
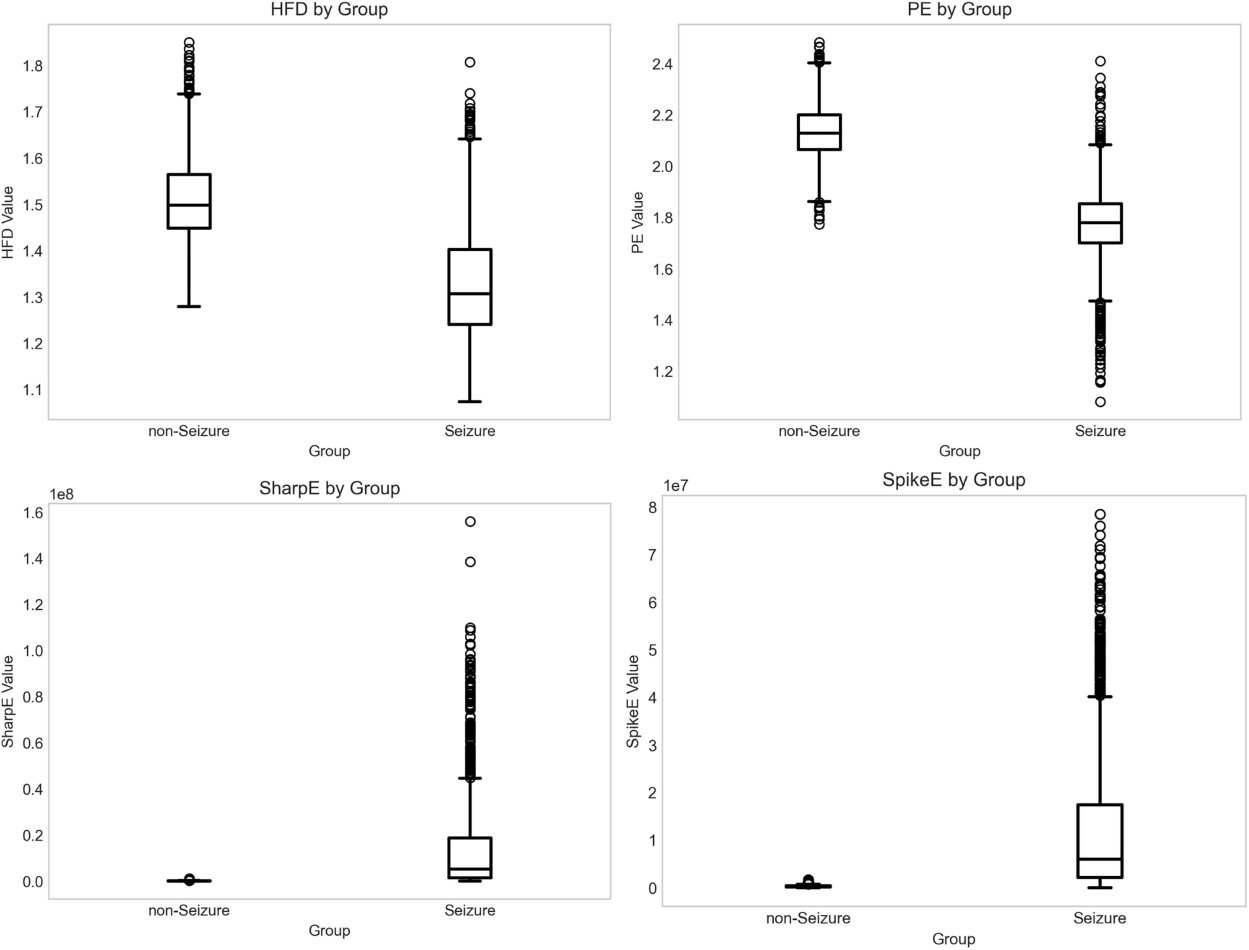
Box plot of the BONN dataset features.

In the box plot analysis of the BONN epilepsy EEG dataset, we concentrated on the disparities in feature distribution between epileptic and non-epileptic states. Fig 3 shows the statistically significant differences in the distribution of each feature between the two groups. It is very clear that the spike waves energy and sharp waves energy are the most discriminative features for distinguishing between epileptic and non-epileptic states, which is consistent with the clinical basis for epilepsy diagnosis. Regarding nonlinear dynamic characteristics, the median HFD in the non-epileptic state was elevated, with a somewhat concentrated data distribution, signifying that the complexity of the EEG signals was maintained at a high and steady level during this condition. Conversely, the median of HFD in the seizure phase group was markedly diminished, exhibiting a comprehensive downward change in distribution. The pattern in the disparities of PE values between the two groups mirrored that of HFD while offering supplementary insights into signal regularity. The median PE in the seizure phase group was much lower than in the non-seizure phase group, and its data distribution displayed comparatively less variability. The decrease in both HFD and PE, two nonlinear dynamic characteristics, generally signifies a reduction in signal complexity, which corresponds with the pathophysiological mechanism of epileptic seizures, where abnormal synchronous discharges from numerous neurons result in simplified and regularized patterns of brain electrical activity.

**Fig 4:**
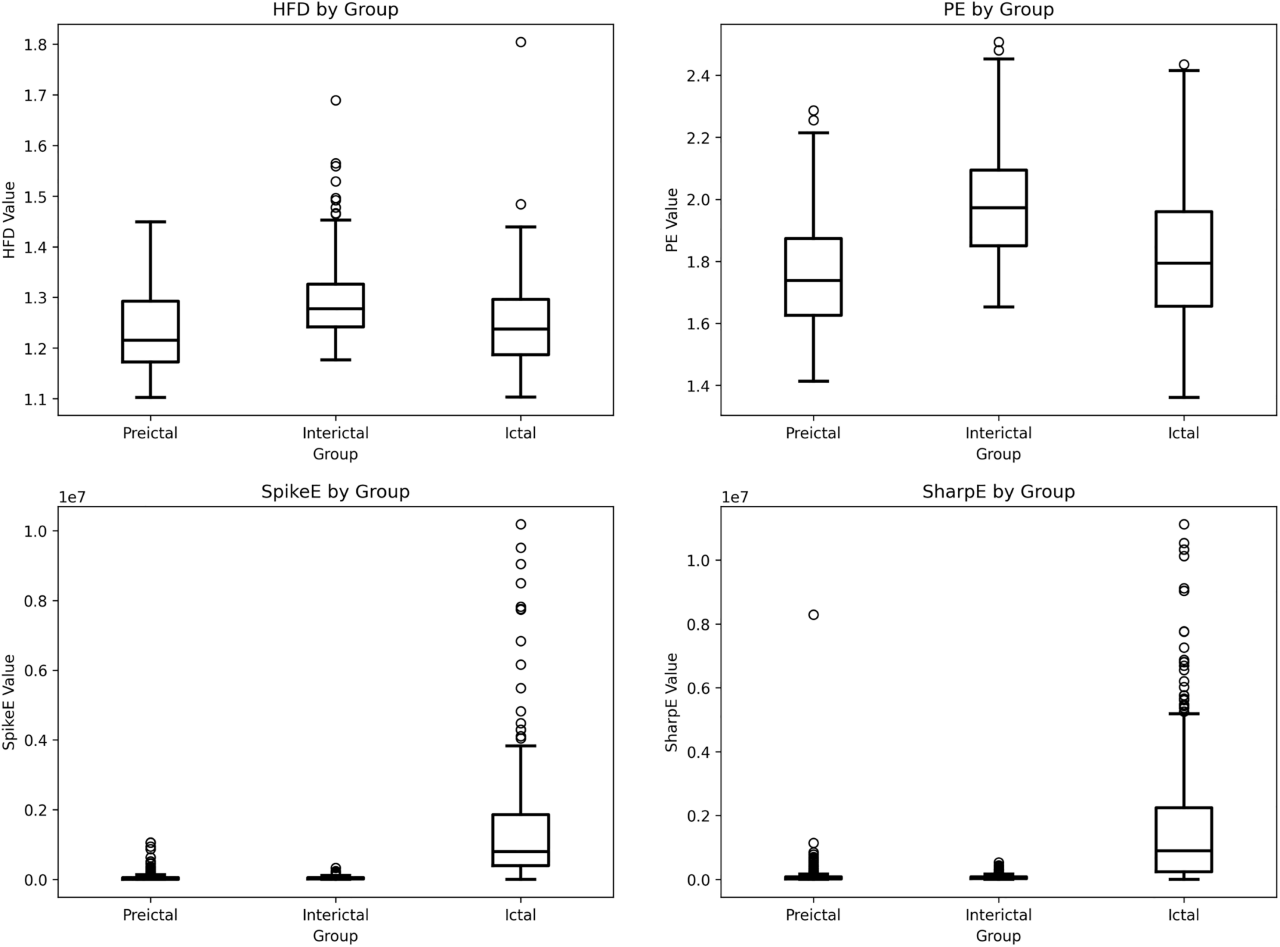
Box plot of features of epilepsy dataset from NDSC.

Unlike the BONN dataset, the epileptic EEG signals from the NDSC have been systematically classified by researchers into three discrete phases: preictal, interictal, and ictal. This dataset enables the investigation of the dynamic progression of brain activity before epileptic episodes. In the box-plot analysis of this dataset, we concentrated on the disparities in feature distributions across the different epileptic EEG phases As shown in Fig 4, the extracted four types of features exhibit statistically significant differences between different EEG states, reflecting good discriminative ability.

Among the two complexity-related features, Higuchi fractal dimension (HFD) and permutation entropy (PE), the interictal state has the highest overall feature values, and the distribution is more centralized and less discrete; while after entering the preictal state, both types of features show a significant downward trend, and the inter-individual differences increase, showing a larger degree of discretization. This phenomenon may reflect the fact that the EEG activity in the pre-seizure stage is in the dynamic process of transition from the stable state to the seizure state, and the closer the data points are to the seizure, the more significant the change of the features, while the signals far away from the seizure stage show a relatively slow change trend, suggesting that the potential value of these two features in the prediction of epileptic seizures.

In terms of pathological characteristics, the BONN dataset exhibits the same features, with higher spike energy and sharp wave energy distribution during the seizure phase. In this dataset, we focus on changes prior to the seizure onset, as shown in the Fig 4. The pathological wave energy in the preictal stage showed a significant upward trend, suggesting that abnormal neural activities at the subclinical level may have begun to appear in the brain at this stage. Through the characterization of different EEG states in the NDSC epilepsy EEG dataset, the changing pattern of electrodynamic features before and after the seizure can be clearly identified. Compared with the analysis of the BONN dataset, the NDSC dataset distinctly captures the evolution of preictal feature distributions, which may offer new insights and could inform the theoretical basis for early epilepsy detection and warning.

Through the box plots we also found that the discretization degree of each type of feature showed significant differences between different categories. This distributional instability may affect the robustness and discriminative ability of the features in the classification task. In order to compensate for the limitations of the original features in portraying state transitions, and to further explore the dynamic evolution of EEG signals in the time dimension, the dynamic feature incremental features proposed in this paper can enrich the feature representation system without destroying the physical meaning of the original features, and also provide more discriminative input variables for the design of the subsequent classifier, which can potentially improve the model’s ability to distinguish between different EEG states. It also provides more discriminative input variables for the design of subsequent classifiers, which may improve the model’s ability to distinguish different EEG states.

### 4.3. Machine learning classification results and analysis

To thoroughly assess the efficacy of the suggested strategy, we developed specific categorization tasks suited to the attributes of each dataset. The Bonn dataset’s main objective was the binary classification of seizure and non-seizure EEG segments, encompassing three comparative experiments: AB vs E, CD vs E, and ABCD vs E. For the NDSC dataset, which offers more detailed temporal annotations, we undertook three specialized tasks: 1) Binary classification of interictal vs ictal phases; 2) Binary classification of preictal vs ictal phases; 3) A more complex multi-class classification encompassing preictal, interictal, and ictal states.

The primary objective of this study is to verify the interpretability of the proposed model and the efficacy of the methodology. Consequently, no parameter optimization was conducted on the classifiers to guarantee consistency and impartiality in the detection results. The primary parameter configurations for the classifiers employed in this investigation are as follows: The radial basis function kernel was selected for SVM, and C was set to 1. The Max_depth was set to 10 for DT, RF, and XGB, and the RF and XGB models included 100 estimators. The KNN classifier was configured with five neighbors. A 10-fold cross-validation technique, stratified at the recording level, was employed to avert data leakage and maintain the integrity of the evaluation. The comprehensive procedure is outlined as follows: Initially, all recordings were divided into 10 segments. In each cross-validation iteration, 9 segments were used for training while 1 segment was retained for testing. Features were retrieved from the windows created from the recordings in the current training fold. Subsequently, normalization parameters were derived exclusively from the characteristics of the training fold and implemented on the features of the matching test fold. The procedure was executed 10 times, and the concluding performance metrics are shown as the mean of the results from the 10 test folds.

Table 1 illustrates the efficacy of the BONN dataset in seizure and non-seizure tasks, highlighting the advantages and applicability of various feature types across different control groups. Pathological characteristics (the energy of spike waves and sharp waves) exhibited remarkable performance across all control groups, attaining accuracies of 96.57%, 94.11%, and 96.84%, respectively, with sensitivities of 96.04%, 93.87%, and 93.1%, demonstrating robust discriminative capability for epileptic conditions. This corresponds with clinical cognition: typical abnormal discharge patterns in epileptic electroencephalograms, such as spike waves and sharp waves, accurately indicate heightened cortical excitability due to their energy fluctuations, rendering them crucial for differentiating between normal and epileptic seizure states. The nonlinear dynamics attribute enhanced the efficacy of the majority of models in the “CD vs E” and “ABCD vs E” classification tasks. This may be ascribed to the fact that, despite the CD control group being in a non-epileptic state, its data were obtained from the intracranial lesion region of epileptic patients, and the EEG signals may still exhibit pathological characteristics. The nonlinear dynamics characteristic can enhance the pathogenic symptoms related to complexity when differentiating from the epileptic state. In the three classification tasks, the integration of the two features yielded the most significant enhancements relative to individual features, achieving ideal accuracy rates of 10.98%, 3.24%, and 7.38%, respectively, along with F1 scores of 12.62%, 4.11%, and 12.25%. The dynamic incremental feature captures nuanced fluctuations in different states at a precise level, enhancing detection performance for both seizure and non-seizure conditions. The integration of pathological waveforms, nonlinear dynamics, and dynamic incremental characteristics markedly enhanced the model’s classification efficacy in the majority of instances.

**Table 1:** Classification tasks for the BONN epilepsy EEG dataset.

AB vs E								
Feature Type			Mould	Evaluation indicators				
PLF	NDF	DFI		Acc	Pre	Sen	F1	Spe
√			DT	0.9546	0.9487	0.9497	0.9491	0.9497
			RF	0.9653	0.963	0.9591	0.9608	0.9591
			KNN	0.964	0.9622	0.9567	0.9593	0.9567
			SVM	0.962	0.9673	0.9477	0.9564	0.9477
			XGB	0.9684	0.9686	0.9604	0.9642	0.9604
	√		DT	0.8504	0.8302	0.8567	0.8389	0.8567
			RF	0.8656	0.8455	0.866	0.8533	0.866
			KNN	0.8544	0.8382	0.832	0.8348	0.832
			SVM	0.8598	0.8415	0.8736	0.8504	0.8736
			XGB	0.8718	0.861	0.8471	0.853	0.8471
√	√		DT	0.9691	0.9653	0.9655	0.9653	0.9655
			RF	0.9809	0.98	0.977	0.9784	0.977
			KNN	0.9669	0.9733	0.953	0.962	0.953
			SVM	0.9722	0.9774	0.9606	0.9682	0.9606
			XGB	**0.9816**	**0.9811**	**0.9775**	**0.9792**	**0.9775**
√	√	√	DT	0.9769	0.9742	0.974	0.974	0.974
			RF	0.9851	0.9842	0.9822	0.9832	0.9822
			KNN	0.942	0.9568	0.9153	0.9319	0.9153
			SVM	0.9722	0.9769	0.961	0.9682	0.961
			XGB	**0.9873**	**0.9878**	**0.9838**	**0.9857**	**0.9838**
**CD vs E**								
**Feature Type**			**Mould**	**Evaluation indicators**				
**PLF**	**NDF**	**DFI**		**Acc**	**Pre**	**Sen**	**F1**	**Spe**
√			DT	0.9251	0.9123	0.9228	0.9169	0.9228
			RF	0.9387	0.9263	0.9394	0.9321	0.9394
			KNN	0.9251	0.9148	0.9178	0.9161	0.9178
			SVM	0.9413	0.9324	0.9367	0.9344	0.9367
			XGB	0.9411	0.9307	0.9387	0.9344	0.9387
	√		DT	0.9093	0.8959	0.9016	0.8986	0.9016
			RF	0.9298	0.921	0.9213	0.921	0.9213
			KNN	0.9251	0.9194	0.911	0.9149	0.911
			SVM	0.9269	0.9144	0.9237	0.9186	0.9237
			XGB	0.9338	0.9283	0.922	0.9249	0.922
√	√		DT	0.9531	0.9457	0.9493	0.9474	0.9493
			RF	0.966	**0.9609**	0.9628	0.9618	**0.9628**
			KNN	0.9593	0.9557	0.9526	0.9541	0.9526
			SVM	0.9602	0.953	0.9583	0.9555	0.9583
			XGB	**0.9662**	0.96	**0.9647**	**0.966**	0.9609
√	√	√	DT	0.9556	0.9481	0.9522	0.966	0.9609
			RF	0.9696	0.9636	0.9686	0.966	0.9686
			KNN	0.9511	0.952	0.9375	0.9441	0.9375
			SVM	0.9631	0.9568	0.9606	0.9587	0.9606
			XGB	**0.9747**	**0.9709**	**0.9723**	**0.9716**	**0.9723**
**ABCD vs E**								
**Feature Type**			**Mould**	**Evaluation indicators**				
**PLF**	**NDF**	**DFI**		**Acc**	**Pre**	**Sen**	**F1**	**Spe**
√			DT	0.934	0.8881	0.9148	0.9003	0.9148
			RF	0.9539	0.9207	0.9393	0.9295	0.9393
			KNN	0.9489	0.9215	0.9187	0.92	0.9187
			SVM	0.953	0.9177	0.9415	0.9287	0.9415
			XGB	0.9557	0.9309	0.931	0.9309	0.931
	√		DT	0.8561	0.7783	0.8514	0.8031	0.8514
			RF	0.8816	0.8089	0.8701	0.8325	0.8701
			KNN	0.8869	0.8289	0.8078	0.8175	0.8078
			SVM	0.8629	0.7897	0.8811	0.8177	0.8811
			XGB	0.9004	0.8562	0.8216	0.8368	0.8216
√	√		DT	0.9589	0.9312	0.9428	0.9367	0.9428
			RF	0.9723	0.9539	**0.9603**	0.957	**0.9603**
			KNN	0.9666	0.9608	0.9334	0.9462	0.9334
			SVM	0.9628	0.9347	0.9521	0.943	0.9521
			XGB	**0.9742**	**0.9626**	0.9562	**0.9593**	0.9562
√	√	√	DT	0.9629	0.9359	0.9505	0.9429	0.9505
			RF	0.9756	0.9604	0.9638	0.962	0.9638
			KNN	0.9536	0.9566	0.8963	0.9225	0.8963
			SVM	0.9541	0.9194	0.9429	0.9304	0.9429
			XGB	**0.9787**	**0.9689**	**0.9643**	**0.9665**	**0.9643**

The results of the multi-tasks classification experiments based on the NDSC epilepsy EEG dataset are shown in Table 2, where different feature types show significant differences in different stages of epilepsy detection. Pathological characteristics demonstrate exceptional efficacy in differentiating interictal from ictal states, as well as preictal from ictal states, with XGB model accuracies of 98.25% and 95.33%, respectively. This indicates that such features adeptly encapsulate the energy fluctuations of abnormal cerebral discharges during seizures, exhibiting a high level of sensitivity and specificity in state discrimination. In the nuanced classification task of preictal, interictal, and ictal states, effective differentiation is challenging when solely depending on pathological or nonlinear kinetic features. The accuracy of XGB is merely 67.2% and 51.3%, indicating the restricted efficacy of these features in identifying subtle state transitions. The effective integration of the two feature types significantly enhances detection performance, with the XGB model achieving accuracies of 17.86% and 33.76%, demonstrating that the fused features can elucidate the nuanced variations in the complexity of the neural network. Moreover, the incremental dynamical features enhance the model’s robustness and generalization capacity across most tasks; specifically, the XGB model attained improvements of 1.08%, 0.66%, and 1.24% in accuracy across three classification tasks when employing the three feature fusion methods. The accuracy has been enhanced to 98.25%, 95.33%, and 86.3%. The F1 scores were enhanced to 98.23%, 95.27%, and 85.79%, respectively. They demonstrate their pivotal significance in encapsulating the trajectory of state evolution.

**Table 2:** Classification tasks for the NDSC epilepsy EEG dataset.

Interictal vs Ictal								
Feature Type			Mould	Evaluation indicators				
PLF	NDF	DFI		Acc	Pre	Sen	F1	Spe
√			DT	0.97	0.9704	0.9708	0.97	0.9708
			RF	0.9684	0.9691	0.9696	0.9683	0.9696
			KNN	0.97	0.9705	0.9711	0.97	0.9711
			SVM	0.9567	0.9589	0.9578	0.9566	0.9578
			XGB	0.9683	0.9688	0.9695	0.9683	0.9695
	√		DT	0.675	0.6806	0.6749	0.6721	0.6749
			RF	0.7067	0.7131	0.7058	0.7037	0.7058
			KNN	0.6817	0.684	0.6817	0.68	0.6817
			SVM	0.7267	0.739	0.7259	0.7223	0.7259
			XGB	0.7017	0.703	0.7017	0.7009	0.7017
√	√		DT	0.9733	0.9735	0.9739	0.9733	0.9739
			RF	**0.9717**	**0.9718**	**0.9726**	**0.9717**	**0.9726**
			KNN	0.9333	0.9414	0.934	0.933	0.934
			SVM	0.95	0.9544	0.9507	0.9498	0.9507
			XGB	**0.9717**	**0.9718**	**0.9726**	**0.9717**	**0.9726**
√	√	√	DT	0.975	0.9752	0.9753	0.975	0.9753
			RF	0.98	0.9805	0.9807	0.98	0.9807
			KNN	0.8683	0.8943	0.8683	0.8659	0.8683
			SVM	0.94	0.9469	0.941	0.9398	0.941
			XGB	**0.9825**	**0.9822**	**0.9831**	**0.9823**	**0.9831**
**Preictal vs Ictal**								
**Feature Type**			**Mould**	**Evaluation indicators**				
**PLF**	**NDF**	**DFI**		**Acc**	**Pre**	**Sen**	**F1**	**Spe**
√			DT	0.9167	0.9178	0.9158	0.9162	0.9158
			RF	0.9333	0.9343	0.9326	0.9329	0.9326
			KNN	0.925	0.9264	0.9238	0.9245	0.9238
			SVM	0.92	0.9218	0.9197	0.9198	0.9197
			XGB	0.9283	0.9304	0.9271	0.9277	0.9271
	√		DT	0.61	0.6115	0.6106	0.6091	0.6106
			RF	0.63	0.6307	0.6296	0.6286	0.6296
			KNN	0.6317	0.6326	0.6311	0.6302	0.6311
			SVM	0.6067	0.6224	0.6067	0.5985	0.6067
			XGB	0.6317	0.6312	0.6312	0.6309	0.6312
√	√		DT	0.9167	0.9199	0.9156	0.9162	0.9156
			RF	0.9417	0.9431	0.9408	0.9412	0.9408
			KNN	0.92	0.926	0.9195	0.9196	0.9195
			SVM	0.925	0.9283	0.9246	0.9247	0.9246
			XGB	**0.9467**	**0.947**	**0.9464**	**0.9465**	**0.9464**
√	√	√	DT	0.905	0.907	0.9039	0.9044	0.9039
			RF	0.9434	0.9458	0.9424	0.9428	0.9424
			KNN	0.8533	0.8733	0.8534	0.8513	0.8534
			SVM	0.9317	0.9339	0.9308	0.9314	0.9308
			XGB	**0.9533**	**0.9527**	**0.9542**	**0.9527**	**0.9542**
**Preictal vs Interictal vs Ictal**								
**Feature Type**			**Mould**	**Evaluation indicators**				
**PLF**	**NDF**	**DFI**		**Acc**	**Pre**	**Sen**	**F1**	**Spe**
√			DT	0.695	0.6802	0.6811	0.6764	0.8463
			RF	0.7087	0.6959	0.6997	0.694	0.8539
			KNN	0.6767	0.6664	0.6708	0.6664	0.8393
			SVM	0.6552	0.5681	0.633	0.537	0.8252
			XGB	0.672	0.6638	0.6641	0.6604	0.8363
	√		DT	0.5134	0.5087	0.5158	0.5054	0.7563
			RF	0.5559	0.5558	0.5603	0.5464	0.7779
			KNN	0.5127	0.5145	0.5192	0.5106	0.757
			SVM	0.5534	0.5792	0.5592	0.5209	0.7784
			XGB	0.513	0.5116	0.5211	0.5083	0.7568
√	√		DT	0.8261	0.8266	0.8219	0.8215	0.9129
			RF	0.861	0.8568	0.8545	0.8533	0.9305
			KNN	0.7884	0.8002	0.7887	0.7888	0.8952
			SVM	0.8033	0.8173	0.7976	0.799	0.9012
			XGB	0.8506	0.8474	0.8474	0.8458	0.9252
√	√	√	DT	0.8311	0.8326	0.8281	0.8278	0.9153
			RF	0.8587	0.863	0.8509	0.8497	0.9288
			KNN	0.7366	0.7645	0.7344	0.7362	0.8689
			SVM	0.796	0.8118	0.7894	0.7919	0.8974
			XGB	**0.863**	**0.863**	**0.8595**	**0.8579**	**0.9314**

**Fig 5:**
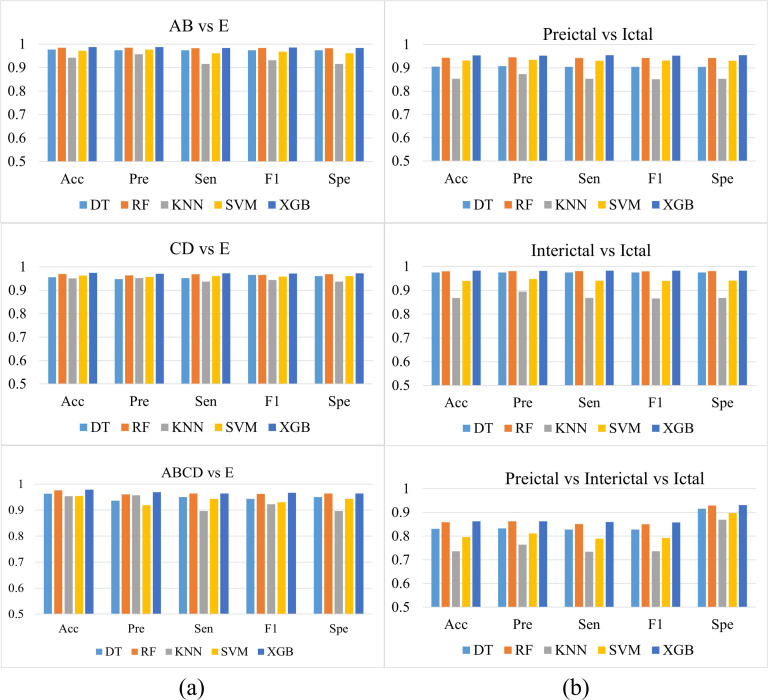
Performance comparison of different classifiers in the epilepsy detection task. (a) Three detection tasks on the BONN dataset; (b) Three detection tasks on the NDSC dataset.

To provide a more intuitive view of the robustness of the proposed model, Fig 5 presents bar charts depicting the classification performance of different classifiers. Panel (a) illustrates the performance comparison of various classifiers on three classification tasks within the BONN dataset, while panel (b) shows the performance comparison of different classifiers on three classification tasks within the epileptic EEG dataset from NDSC.

The bar charts comparing the detection performance of the two datasets show that the performance of the different classifiers is highly similar. In the three classification tasks of the BONN dataset, the standard deviation range of each evaluation metric on different classifiers is 0.01–0.03; in the corresponding tasks of the NDSC dataset, the standard deviation range is slightly larger, 0.03–0.05. This smaller standard deviation range adequately suggests that the difference in performance between different classification models is not statistically significant. This finding further validates that the feature extraction method proposed in this study has excellent discriminative ability in the epilepsy detection task, and its effectiveness is minimally affected by the significant influence of the specific classifier selection, with good generalization ability.

Combining the classification tasks of the above two datasets, some common patterns can be found, i.e., pathological features are the most discriminative for seizure status, which are consistent with clinical diagnostic criteria and highly interpretable. Nonlinear features enhance the ability to perceive subtle neural activity changes, especially in the preictal period, which is consistent with the fact that the brain has already shown subclinical manifestations prior to the somatization response, and is a very effective features, while the dynamic incremental features provide evolutionary information in the time dimension. The combination of the three constructs a multi-level and multi-angle epilepsy detection feature system from the perspective of static and dynamic features, and from the perspective of multidomain fusion, providing a methodological basis for realizing a high-precision and high-robust automatic identification system. It is fully proven that our proposed features are highly competitive.

**Fig 6:**
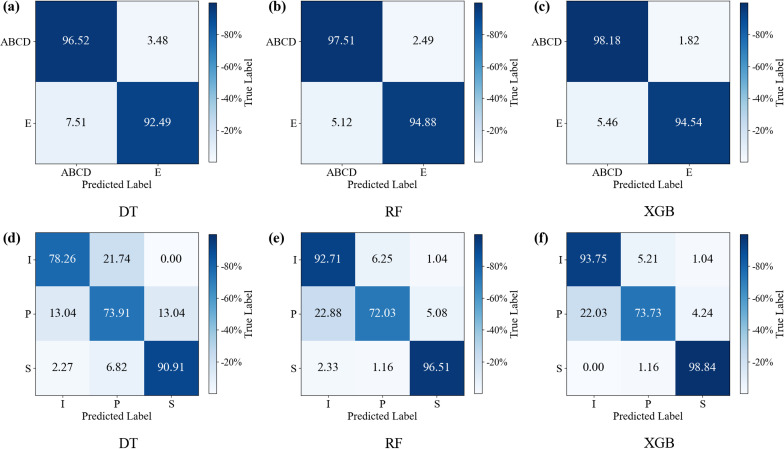
Confusion matrix of models in two datasets detection tasks. (a)~(c) results of different classifiers on ABCD-E classification task; (d)~(f) results of different classifiers on interictal vs preictal vs ictal.

To thoroughly analyze the classification error distribution characteristics of the proposed method and validate its classification robustness and feature discriminative power, this study selected the most challenging classification tasks from two datasets: the ABCD vs E control group on the BONN dataset and the preictal (P) vs interictal (I) vs ictal (S) control group on the NDSC dataset. The confusion matrices for the top three performing models are analyzed, as shown in Fig 6. The rows and columns of the matrix correspond to true labels and predicted labels, respectively. All classifiers exhibit highly consistent patterns in distinguishing non-seizure (ABCD) from seizure (E) periods. The non-seizure correct recognition rate consistently remains above 96.52%, with only a small number misclassified as seizure. The seizure correct recognition rate also stably maintains above 92.49%, and the misclassification rate for both categories remains consistently low. For the NDSC dataset, the confusion matrix reveals that all classifiers maintain high recognition accuracy for seizure periods and equally stable recognition for interictal periods. The primary source of error significantly stems from misclassifications of samples in the preictal and interictal phases. This aligns perfectly with clinical reality, demonstrating both the features’ precise capture of physiological differences across phases and their interpretability.

### 4.4. Feature interpretability analysis

Interpretability makes models transparent and trustworthy, and the interpretability provided by these techniques helps to trust the decisions made by automated models. Among them SHAP provides a way to analyze the contribution of each feature to the total model prediction. From the experiments, it can be seen that the XGB classifier achieves the highest classification performance across all six tasks. Therefore, we plotted the XGB based SHAP plot.

**Fig 7:**
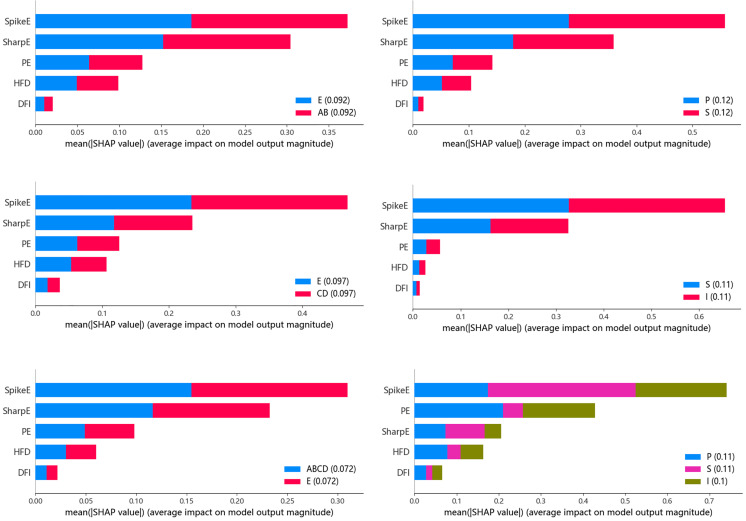
SHAP plot analysis of the BONN dataset and NDSC dataset. (a) BONN dataset (b) NDSC dataset, I stands for interictal, P for preictal, and S for ictal.

Fig 7 shows the SHAP plots for the three classification tasks of the BONN dataset and NDSC. SHAP value analysis results indicate that across all epilepsy vs. non-seizure classification tasks in both datasets, two pathological features consistently emerged as the most critical indicators for distinguishing epileptic seizures, demonstrating exceptional discriminatory power. Nonlinear dynamical features characterize different states of EEG signals from a physiological perspective, such as complexity and regularity, playing a particularly crucial role in the most challenging three-classification task: distinguishing between preictal, interictal, and ictal periods. These features effectively reflect the randomness and complexity of EEG signals, providing key quantitative evidence for epilepsy detection. Additionally, the Dynamic Feature Increment (DFI) offers valuable supplementary information across all tasks. SHAP interpretability analysis corroborates the machine learning-based detection results.

**Fig 8:**
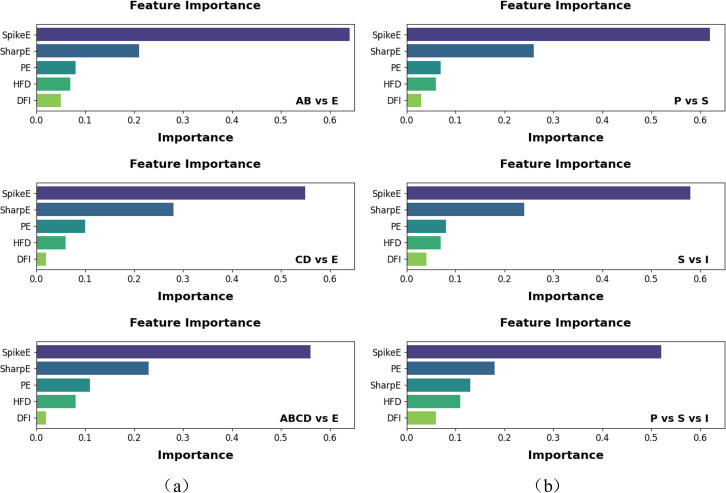
Feature importance analysis of epilepsy dataset from BONN and NDSC. (a) BONN dataset (b) NDSC dataset, I stands for Interictal, P for Preictal, and S for Ictal.

To further illustrate the strong reliability of our interpretability analysis, we concurrently plotted feature importance analysis based on XBG classification. Fig 8 illustrates that, with the exception of the CD vs E control group, the feature importance rankings for all other classification tasks are in complete concordance with those derived from the SHAP-based interpretability study. Within the CD versus E control group, sharp waves energy is just slightly superior to spikes wave energy, exhibiting low variation in the SHAP analysis. This repeatedly illustrates that pathological characteristics exert complete dominance in both seizure and non-seizure classification tests. Collectively, these findings not only validate the representational power of the selected features but also confirm that the effective integration of pathological and nonlinear dynamical features significantly enhances the overall performance of epilepsy detection.

### 4.5. Results and analysis of cross-subject experiments

This work conducted focused cross-dataset validation studies to assess the generalization capability of the proposed feature combination and model. To enhance the uniformity of EEG signal attributes, the ABCD versus E control group from the BONN dataset and the Interictal versus Ictal control group from the NDSC dataset were chosen, employing the “single dataset training and another dataset testing” methodology for cross-dataset experimentation. The findings are presented in Table 3. Utilizing the BONN dataset with an expanded sample size for training and the NDSC dataset for testing, the XGB model achieves a classification accuracy of 82.17% an F1 score of 81.14%, while maintaining a balance in sensitivity and specificity. Utilizing the NDSC dataset with a reduced sample size for training resulted in a notable loss in the model’s metrics on the BONN dataset: the accuracy decreased to 77.17%, and the F1 score fell to 77.13%. Despite the absence of substantial divergence among the criteria, the total performance was marginally inferior to the previous one. Although model performance decreased following cross-dataset transfer relative to validation inside a single dataset, it remained substantially superior to random guessing. This outcome demonstrates that the feature combination suggested in this study can proficiently identify the shared discriminative characteristics of EEG signals during ictal and interictal phases, remaining largely unaffected by variations in dataset origins and patient demographics, thereby possessing notable cross-data applicability. It also confirms that the model is not too tailored to the unique characteristics of a singular dataset.

**Table 3:** Results of the cross-dataset epileptic seizure detection.

Trainset	Testset	Acc(%)	Pre(%)	Sen(%)	F1(%)
BONN	NDSC	82.17	83.46	82.17	81.14
NDSC	BONN	77.17	77.33	77.17	77.13

### 4.6. Comparative experimental results and analysis

In order to validate the effectiveness of the proposed method in this study, comparative experiments are conducted between the proposed model and the latest epilepsy detection methods, which include feature extraction-based machine learning methods, feature extraction-based deep learning methods, and end-to-end deep learning-based methods, aiming to highlight the advancement of the proposed model while making a comprehensive comparison (Table 4).

**Table 4:** Comparison of the latest methods on the BONN dataset.

Categorization	Source	Number of Features/ Feature Filtering	Classification method	Acc(%)
AB-E	Chen et al. [14]	13/yes	GA-BP	97.67
	Al-Hadeethi et al. [15]	10/ yes	KST + AdaBoost	98.38
	Qureshi et al. [16]	14/ yes	FNN	90
	Wang et al. [17]	-/-	CNN+LSTM	98.3
	**This Work**	**5/no**	**XGB**	**98.73**
CD-E	Chen et al. [14]	20/ yes	GA-BP	97.47
	Piyush Swami et al. [30]	42/no	GRNN	95.2
	Qureshi et al. [16]	14/ yes	FNN	91.11
	Yilmaz Kaya et al. [31]	-/-	LBP+BayesBet	97
	Jiang et al. [32]	-/-	ED-TSK-FC	97.14
	**This Work**	**5/no**	**XGB**	**97.47**
ABCD-E	Yatindra Kumar et al. [33]	6/no	SVM	94
	Chen et al. [14]	20/ yes	SVM	96.33
	Chen et al. [14]	20/ yes	GA-BP	97.16
	**This Work**	**5/no**	**XGB**	**97.87**

From the table, we can find that the existing epilepsy detection methods for feature extraction and machine learning have a large number of selected features, Chen et al extracted 13 traditional features from different wavelet subbands, Al-Hadeethi et al extracted 10 basic features, and Qureshi et al. even extracted 14 features, and these methods need to rely on the feature selection methods to screen the extracted features with effective features to achieve better detection performance, too many features will bring information redundancy, after feature screening will increase the complexity of the detection model, and the proposed features are mostly based on the statistical properties of the data, and the medical interpretability is poor. With the rapid advancement of deep learning technology, numerous scholars have applied it to epilepsy detection research. For instance, Wang et al. proposed an end-to-end detection method combining CNN and LSTM, achieving 98.3% accuracy in the AB vs E control group of the BONN dataset; Jiang et al. achieved 97.14% accuracy in the CD vs. E control group using a TSK fuzzy classifier. However, despite their superior performance, deep learning models inherently suffer from complex structures and poor interpretability. The method proposed in this paper utilizes only five physiologically interpretable features, achieving excellent performance across different classifiers without requiring feature selection. Notably, across all detection tasks based on the XGB classifier, accuracy ranged from 97.47% to 98.73%, representing a maximum improvement of 1.06% over traditional machine learning models and 8.73% over deep learning detection models. The proposed epilepsy detection model breaks the conventional paradigm that high performance detection must rely on deep learning or complex feature engineering, providing a more transparent foundation for clinical decision-making tasks.

## 5. Conclusions

This study tackles the shortcomings of conventional epileptic EEG detection techniques, including inadequate model interpretability and restricted generalization ability, by introducing an interpretable low-dimensional feature amalgamation and a machine learning framework grounded in physiological mechanisms, thus offering a novel methodology for automated epilepsy diagnosis. This study’s feature combination, based on the physiological properties of epileptic convulsions, encompasses pathogenic features, nonlinear dynamic features, and their dynamic increments. This method not only corresponds with the pathophysiological nature of EEG signals but also mitigates model redundancy resulting from intricate features. This feature combination exhibits stable and consistent classification performance across multiple public datasets using five widely utilized classifiers: SVM, RF, DT, KNN, and XGB. This indicates robust discriminative power and broad applicability, showing no significant dependence on specific models, thereby offering flexibility for model selection in various application contexts. This study employed the Shap interpretability analysis approach to examine the decision-making logic of the model, elucidate the contribution weights of each feature across several seizure stages of epilepsy, and align the model’s predictive outcomes with clinical pathological understanding. The cross-dataset validation results indicate that while model performance is slightly diminished relative to a single dataset, it sustains a high overall detection rate, confirming the robustness of the feature combination and the model’s cross-domain adaptability, thereby offering dependable support for clinical applications.

The study possesses specific limitations. The free datasets included in this research exhibit limited sample sizes and are primarily comprised of brief EEG segments, lacking validation against extensive, continuous, long-term EEG data. Subsequent research should assess model efficacy using extensive long-term EEG datasets. The data segmentation strategy necessitates optimization. The fixed-length segmentation method utilized in this study may induce border effects between physiological states and does not adequately address the dynamic, time-varying properties of EEG signals. Shorter segments may hinder the detection of low-frequency features, whereas longer segments could hide the nuances of localized aberrant discharges. Future research should investigate adaptive segmentation techniques initiated by physiological events to improve the specificity and precision of feature extraction.
